# First report of a parapoxvirus red deer infection in reindeer (*Rangifer tarandus tarandus*): clinical presentation and full-genome characterization

**DOI:** 10.1186/s12985-025-03046-5

**Published:** 2025-12-31

**Authors:** Karin Wallin Philippot, Mikael Leijon, Ylva Lindgren, Fereshteh Banihashem, Tomas Jinnerot, Veronica Lengquist, Faisal Suhel, Cathrine Arnason Bøe, Bjørn Spilsberg, Ingebjørg Helena Nymo, Ulrika Rockström, Jonas Johansson Wensman

**Affiliations:** 1 Department of Clinical Sciences, Faculty of Veterinary Medicine and Animal Science, Swedish University of Agricultural Sciences, P.O. Box 7054, 750 07 Uppsala, Sweden; 2 Department of Animal Health and Antimicrobial Strategies, Swedish Veterinary Agency, 751 89 Uppsala, Sweden; 3 Department of Microbiology, Swedish Veterinary Agency, 751 89 Uppsala, Sweden; 4 Department of Pathology and Wildlife Diseases, Swedish Veterinary Agency, 751 89 Uppsala, Sweden; 5 Department of Animal Biosciences, Faculty of Veterinary Medicine and Animal Science, Swedish University of Agricultural Sciences, P.O. Box 7023, 750 07 Uppsala, Sweden; 6 Department of Analysis and Diagnostics, The Norwegian Veterinary Institute, Ås, Norway; 7 Research Group Food Safety and Animal Health, The Norwegian Veterinary Institute, Tromsø, Norway; 8 Department of Arctic and Marine Biology, Faculty of Biosciences, Fisheries and Economics, UiT, The Arctic University of Norway, Tromsø, Norway; 9 Farm & Animal Health, Uppsala, Sweden

**Keywords:** Eurasian tundra reindeer, Full genome, Host adaptation, Parapoxvirus, P*arapoxvirus reddeerpox*, RDPV

## Abstract

In September 2023, five semi-domesticated reindeer calves (*Rangifer tarandus tarandus*) from Norrbotten County, Sweden, displayed clinical signs resembling contagious ecthyma. Samples were collected from the lesion sites, including the skin of the muzzle, eyelids, and oral mucosa. The samples were analysed via real-time PCR (qPCR) for parapoxviruses (PPV) and cervidpoxviruses (CvPV). Both viruses were detected in samples from several calves. Full genome sequencing of the PPV strain 23-MIK191411 revealed close similarity to the parapoxvirus red deer (RDPV) strain HL953 from a red deer (*Cervus elaphus*) in Germany but with a novel 2,187 bp insert in the right terminal third of the genome. This insert carried two open reading frames (ORFs) that, while divergent, presented sequence homology with several other PPVs. Analysis of amino acid differences relative to the sequence of the RDPV HL953 strain revealed that proteins implicated in host interactions and virulence presented the greatest differences. Thus, comparative sequence analysis indicates that an RDPV has recombined with an ancestor of 23-MIK191411 and adapted to the reindeer host. This study represents the first detection of RDPV, with a unique insert likely originating from an unknown PPV in reindeer, indicating its emergence beyond the red deer host range. Clinical signs are consistent with those caused by other poxviruses, including CvPV also detected in the investigated animal group, making it difficult to diagnose the causative agent solely via clinical observation. In the sequenced sample, RDPV was confirmed as the predominant variant. These findings underscore the importance of molecular diagnostics for accurate pathogen identification and highlight the need for continued health surveillance of reindeer. Further investigations are needed to determine the clinical impact of RDPV in reindeer.

**Supplementary Information:**

The online version contains supplementary material available at 10.1186/s12985-025-03046-5.

## Introduction

The genus of parapoxviruses (PPVs), a member of the family *Poxviridae* and subfamily *Chordopoxvirinae*, causes contagious pustular dermatitis and stomatitis in various host species. This genus is currently divided into five recognized species: *Parapoxvirus orf* (orf virus, ORFV), *Parapoxvirus bovinestomatitis* (bovine papular stomatitis virus, BPSV), *Parapoxvirus pseudocowpox* (pseudocowpox virus, PCPV), *Parapoxvirus sealpox* (seal parapoxvirus, GSEPV), and *Parapoxvirus reddeerpox* (parapoxvirus red deer, RDPV) [1]. In addition, a related, unclassified horse parapoxvirus was recently isolated from horses with dermatitis in Finland [2, 3]. All PPVs are known or considered zoonotic, although the host range for the specific PPV species varies. ORFV is considered to have a broad host range and is distributed worldwide, causing contagious ecthyma (CE) (*syn.* contagious pustular dermatitis, scabby mouth) in domestic sheep and goats. Notably, ORFV has also been detected in naturally infected domestic and wild ruminants, such as reindeer, caribou, muskoxen, Alaskan mountain goats, and camelids [4–7]. Bovine papular stomatitis virus and PCPV infect mainly cattle, and RDPV has previously not been detected in species other than red deer (*Cervus elaphus*) [4, 8, 9].

Clinical signs of RDPV, mainly scabs on velvet antlers, ears, muzzles, and lip margins, were first described in farmed red deer in New Zealand (NZ) in 1985–1986 [10]. Red deer were imported to NZ from Europe in 1851, and just before the first reports of clinical signs, red deer farming had undergone a period of intensification [9]. The first report of RDPV outside of NZ was in wild red deer with severe stomatitis in Italy from 2008–2009. The virus is closely related to the RDPV found in NZ [11]. In Germany, a similar virus was found in tonsil swabs from apparently healthy red deer hunted in the Bavarian Alps, confirming RDPV as a separate species within the PPV genus [8]. In addition, the results suggest that subclinical infection in animals could play a role in disease dynamics. Sheep experimentally infected with RDPV have only displayed mild clinical signs, similar to lesions induced by ORFV [4, 9].

Chordopoxviruses generally consist of a core region of 88 well-conserved genes [12, 13]. Genus- or species-specific genes are located outside the core region and are of particular importance to virulence and virus‒host interactions. To date, only two isolates of RDPV, RD86 and HL953, have been successfully propagated in cell culture, and only the full genome of RDPV HL953 is available [8, 9].

In semi-domesticated reindeer (*Rangifer tarandus tarandus*), ORFV and PCPV have been described as the causative agents of outbreaks in Fennoscandia [7, 14–17]. Clinical signs include severe crusty lesions, especially on the muzzle and in the oral mucosa. These lesions may lead to secondary bacterial infections, impairing the ability to eat, and consequently, starvation and mortality [7, 14, 16, 18–20]. In addition, a cervidpoxvirus (CvPV) was first described in semi-domesticated reindeer from Norway and Sweden in September 2023, inducing somewhat similar clinical signs as PPV, such as crusty lesions around the eyes and genitalia [21].

Infectious diseases in reindeer are driven by multiple factors, including climate change and the loss of grazing land due to habitat fragmentation [22, 23]. These developments have increased the need for supplementary feeding, leading to intensified husbandry practices, increased animal density, and stress [24]. Cumulatively, these changes increase the exposure and susceptibility of reindeer to pathogens and exacerbate the spread of existing diseases [18, 24–27].

The occurrence of RDPV-associated disease in semi-domesticated reindeer in Sweden, as described in this study, exemplifies cross-species transmission and viral host adaptation. Moreover, reindeer may serve as reservoirs for zoonotic parapoxviruses, with potential implications for both reindeer health and humans in close contact. These findings underscore the need for continued health surveillance in reindeer populations. This study aimed to document the clinical signs associated with RDPV and to genetically characterize the virus at the full-genome level to enhance the understanding of its origin and potential implications for reindeer health.

## Methods

###  Study herd and sampling of animals

The diseased reindeer came from a reindeer herd consisting of approximately 2,000 reindeer herded year-round in the taiga region of Norrbotten County, Sweden. The herders performed winter feeding on natural pastures when needed. The herd was assembled multiple times per year: during summer for calf earmarking, in autumn for the selection of adult males for slaughter, and from November to December, primarily for the slaughtering of calves. Animals showing clinical signs of disease or any health issues, including signs of eye diseases, were consistently sorted for slaughter, a practice the herders believed to have long-term positive effects on herd health.

Historically, three red deer farms were located within the pastureland of the herding district, overlapping with reindeer grazing areas. These farms were phased out ten to twenty years ago; however, one farm had been repurposed for sheep farming. For several years, herders observed crusts and skin lesions on the heads of individual calves, yearlings, and some adult reindeer, including a castrated male, particularly affecting the muzzle and eyelids. With experience, herders have become more proficient in identifying lesions. However, crusts and lesions in the skin around the eyes and muzzle were believed to be associated primarily with heavy insect activity from July to September.

During a herd gathering in September 2023, ten out of approximately 150 reindeer were observed with skin lesions and crusts on the head, primarily on the muzzle and eyelids. Five calves were isolated and examined further. The calves were sampled by gently rubbing an eSwab™ (Copan Diagnostics, Brescia, Italy) under and on crusts; on lesions on the muzzle (Calf 2 and 5); over the conjunctival fornix of eyes with affected eyelids (Calves 1, 3, 4 and 5); and on lesions in the oral cavity (Calf 5) (Table 1). Sampling was conducted in accordance with institutional and national guidelines (see Ethics statement). Calf 5 was the most severely affected animal with an affected general appearance and was therefore euthanized and necropsied. Tissue samples from the eyelids, oral mucosa, muzzle, spleen, and one submandibular lymph node were collected from Calf 5. Hygienic precautions were implemented to prevent cross-contamination between animals and from the environment. Disposable gloves were changed between each animal, and all samples were collected and handled individually in sterile containers. The samples were packed in separate bags and sent at 4 °C to the Swedish Veterinary Agency (SVA) and the Norwegian Veterinary Institute (NVI).

**Table 1:** Results from real-time PCR analysis for parapoxvirus (PPV) and cervidpoxvirus (CvPV) in five reindeer calves

Calf ID (swabbed lesion site)	PPV, (Ct value)	CvPV, (Ct value)
1 (conjunctiva)	Positive (34.0)	Negative
2 (muzzle)	Negative	Positive (23.8)
3 (conjunctiva)	Negative	Positive (35.8)
4 (conjunctiva)	Negative	Positive (23.3)
5 (conjunctiva)	Positive (19.6)	Positive (29.2)
5 (oral mucosa)	Positive (18.4)	Positive (34.9)

Ct = cycle threshold. A clear curve with a Ct value ≤ 40 was considered positive

### Histopathology

Histopathology was performed on tissue samples from Calf 5. The tissue samples were fixed in 10% neutral buffered formalin, embedded in paraffin, cut at approximately 4 µm, and mounted on glass slides. All the tissue sections were stained with hematoxylin and eosin (H&E) and coverslipped in an automated Dako Coverstainer (Agilent Technologies, Santa Clara, CA, USA). The recommended standardized staining protocol from the vendor was used.

### Real-time polymerase chain reaction

Nucleic acids were extracted from swab samples collected from lesion sites on the five calves via the IndiMag Pathogen Kit (INDICAL BIOSCIENCE GmbH, Leipzig, Germany) and subsequently analysed via real-time PCR (qPCR) for the presence of PPV and CvPV as previously described [21, 28]. For PPV, the forward primer PPV up 5’-TCGATGCGGTGCAGCAC-3’, reverse primer PPV do 5’-GCGGCGTATTCTTCTCGGAC-3’, and the probe PPV TMGB 5’-FAM-TGCGGTAGAAGCC-MGB-3’ targeting the B2L gene were used [28]. For CvPV, forward primer reVir-F 5’- TCTCCAACCATTCCCTGAAC-3’, reverse primer reVir-R 5’- GGTGCRCCTGTAAGAAAGAG-3’, and probe reVir-pr 5’-FAM- ACCATTTGTTACCGCTTGCA-BHQ1-3’ targeting the virion core protein gene were used [21]. In brief, total nucleic acids from 0.2 ml of swab mixture were extracted and eluted in 0.1 ml of AVE buffer according to the manufacturer’s instructions. Real-time PCR for PPV was performed with PerfeCTa qPCR Toughmix (Quantabio, Beverly, MA, USA) in a total reaction volume of 15 µl using 2 µl of sample extract with 0.5 µM of each primer and 0.1 µM of the probe. Real-time PCR for CvPV was performed with Brilliant III Ultra-Fast QPCR Master Mix (Agilent) using 0.9 µM of each primer and 0.6 µM of the probe and 5 µl of sample. Both PCRs were run for 45 cycles with annealing at 60 °C. A clear curve with a cycle threshold (Ct) value ≤ 40 was considered positive. Both qPCR assays have been validated and show no cross-reactivity [21].

### Next-generation sequencing and bioinformatics

Library preparation and metagenomics were carried out at the Clinical Genomics Stockholm facility at the Science for Life Laboratory (Stockholm, Sweden), where 96.5 M 2 × 150 nucleotide paired-end reads were acquired via a NovaSeq X sequencer (Illumina, Inc., San Diego, CA, USA). The paired-end reads were quality trimmed via Trimmomatic v. 0.39 [29] with a sliding window of four nucleotides, and an average quality score of 15 was obtained. The trimmed reads, including singletons, were assembled via SPAdes v. 3.15.4 in standard mode [30]. The assembled contigs were then classified via DIAMOND v. 2.0.9 [31] with the NCBI nr database of GenBank release 248 and the corresponding NCBI taxonomy databases. The last 384 nucleotides of RDPV HL953 (accession NC_025963.1) were identical to the inverted repeat of positions 670–1053 of the SPAdes de novo assembled contig obtained from the strain 23-MIK191411 data. Inverted repeats are expected at the termini of PPVs, but short-read assembly is unable to account for this because all reads originating from the end repeats will end at a single end of the contig. Therefore, the following approach was used. The de novo assembled contig was merged with the last 384 nucleotides of NC_025963.1. Read mapping was then carried out with random selection if a read mapped to more than one site. This ensured equal mapping to both ends. Finally, a consensus sequence was extracted from the map and subjected to the criterion that at least 10 reads should cover each position. A coverage of less than 10 × was indicated with an 'N' in the sequence in the interior and was cropped if it was at the end of the sequence. This procedure, as well as the detection of inverted repeats, was carried out with CLC Genomics Workbench v. 24.0.1 (QIAGEN, Aarhus, Denmark). Annotation was carried out with PROKKA v1.14.5 [32] via RDPV HL953 with the GenBank accession NC_025963.1 as a reference. In a few cases, in which there were alternative start codons, the prediction of PROKKA and the open reading frame (ORF) given in NC_025963.1 differed, and a longer ORF was selected.

### Phylogenetic analysis

The parapoxviruses (with GenBank accession shown within parentheses), RDPV (NC_025963), reindeer RDPV (PV021465), BPSV (NC_005337), PCPV (NC_013804), ORFV (NC_005336), and GSEPV (NC_035188) were subjected to maximum likelihood phylogeny analysis using the CLC Genomics Workbench. Ten parapoxvirus core proteins (the denotation of the Vaccinia virus Copenhagen strain homologues shown within parentheses): poly-A polymerase catalytic subunit (E1L), DNA polymerase (E9L), RNA polymerase associated protein RP94 (H4L), RNA polymerase subunit RPO132 (A24R), IMV protein VP55 (H3L), DNA topoisomerase type 1 (H6R), EEV envelope phospholipase (F13L), serine/threonine kinase (F10L), DNA helicase (A18R), and RNA helicase (I8R) were individually aligned and then concatenated before being subjected to maximum likelihood calculation using a neighbour‒joining starting tree with the WAG protein substitution model. Four substitution rate categories were used. The gamma distribution parameter was set to 1. The bootstrap analysis utilised 1000 replicates.

The same parapoxviruses listed above were also used to carry out recombination analysis using the RDP software version 5.76 beta [33]. The full viral genome sequences were aligned with the CLC genomic workbench, and the alignment was subsequently analysed by RDP5 using the default set of analyses.

## Results

### Clinical presentation

**Fig. 1:**
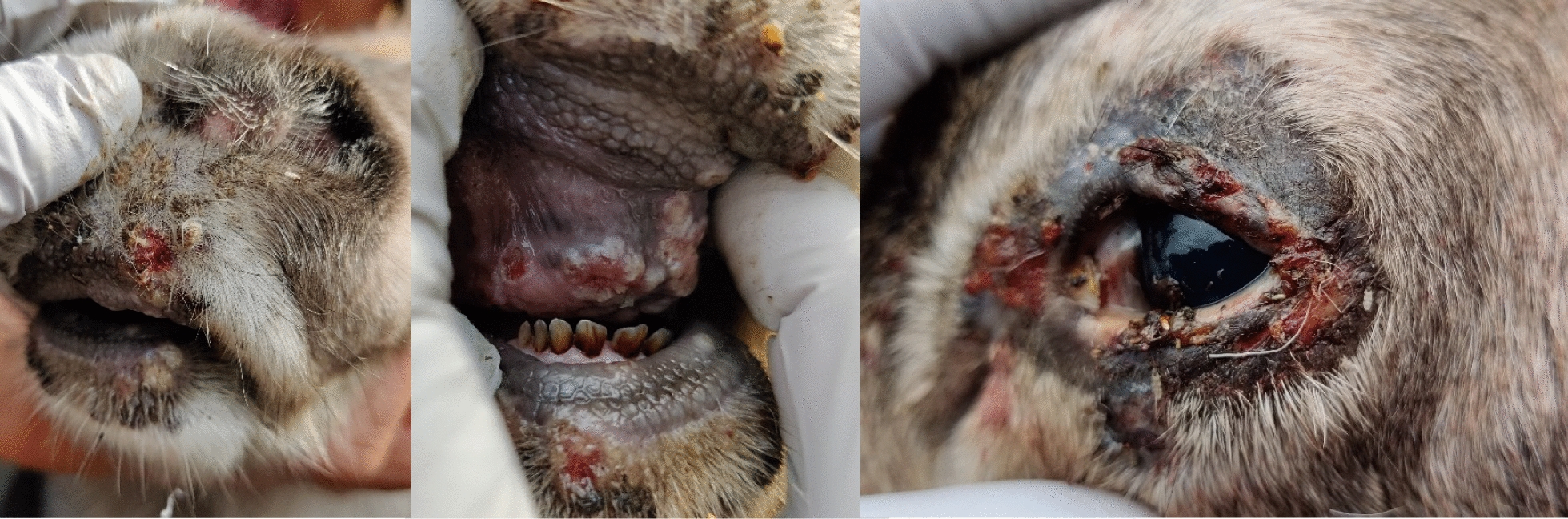
Crusts and proliferative lesions on the muzzle, in the oral cavity, on mucous membranes, and on both eyelids of the most severely affected calf (Calf 5) among five isolated semi-domesticated reindeer calves (*Rangifer tarandus tarandus*). The animals originated from a gathering of approximately 150 reindeer in September 2023 in Norrbotten County, Sweden. Photo: Veronica Lengquist, SLU

During a gathering in September 2023 to select adult males for slaughter, several reindeer calves displayed clinical signs of disease. Ten out of approximately 150 reindeer were observed to have multiple skin lesions and crusts on the head, primarily on the muzzle and eyelids, and some exhibited ocular discharge. Five calves with the most extensive clinical signs were isolated for clinical examination and sampling. All these calves exhibited multiple crusts and skin lesions around one or both eyes, the muzzle, or elsewhere on the head. Three calves (Calves 1, 3, and 4) had mild to moderate lesions on the eyelids of a single eye, crusts with pus, and moderate epiphora. Calf 2 had a focal lesion on the muzzle, one lesion dorsally from the muzzle, and no eyelid lesions (Supplementary Fig. 1). Calf 5 appeared generally affected upon examination, was euthanized and necropsied, and an oral mucosal swab was later selected for sequencing. Multiple crusts and lesions were observed on the eyelids around both eyes, on the muzzle, and on the lips. Similar and proliferative lesions were detected in the oral cavity on mucous membranes, including the hard palate, tongue, and gingiva (Fig. 1). In addition, one submandibular lymph node was mildly enlarged with hyperemic areas. The body condition was normal.

### Histopathology of tissue samples from Calf 5

**Fig. 2:**
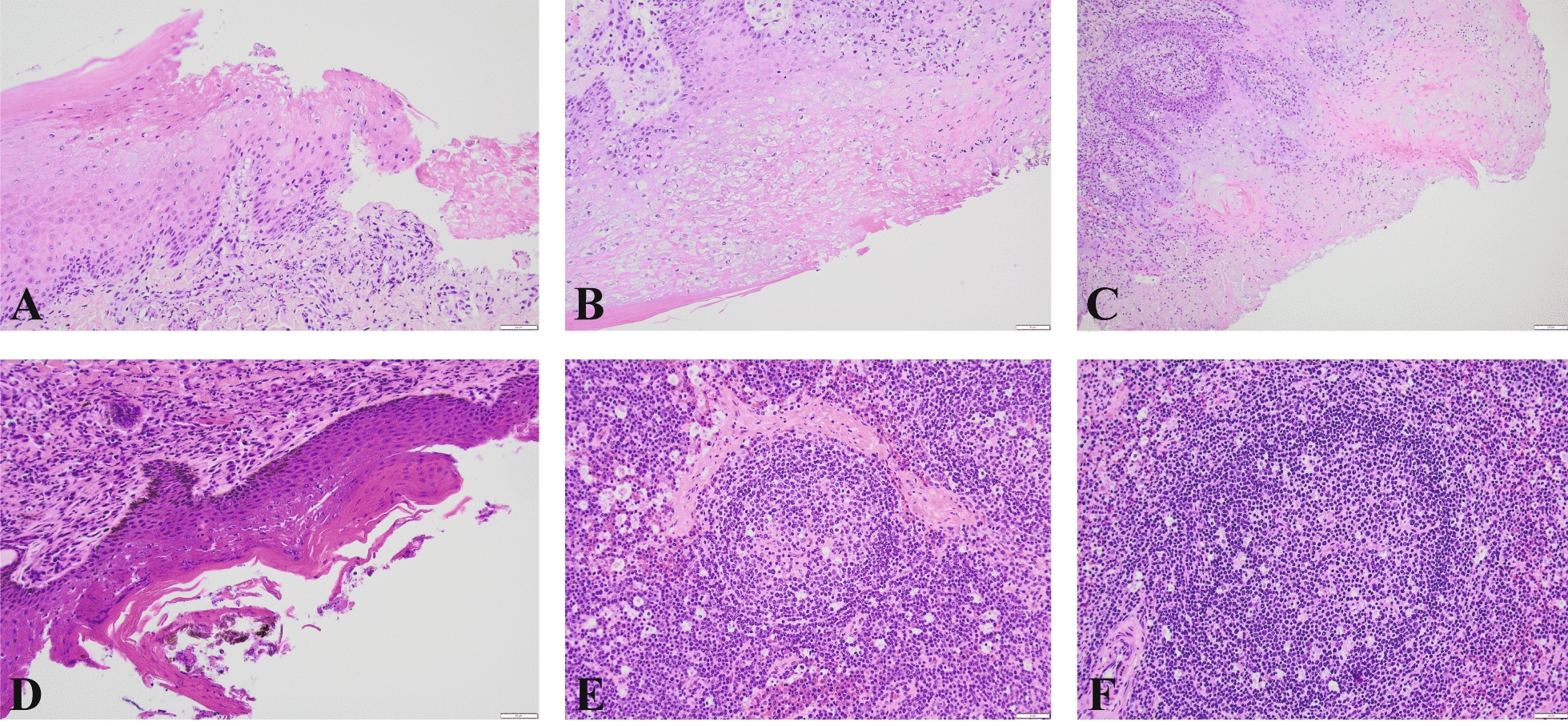
Histopathological findings in tissue samples from the most severely affected reindeer calf (Calf 5). The samples were fixed and stained with hematoxylin and eosin (H&E). Scale bar = 50 µm unless otherwise indicated. **A** Ulceration in the epithelium, with swollen and necrotic keratinocytes, in the oral cavity. **B** Necrotic area in the epidermis of the muzzle. The presence of inclusion bodies was not confirmed but cannot be entirely excluded. **C** Hyperplastic epidermis (to the right) of the muzzle with swollen, degenerated keratinocytes and necrosis. Infiltration of inflammatory cells in the epidermis and dermis. Scale bar = 100 µm. **D** Hyperkeratotic epidermis and infiltration of inflammatory cells in the underlying dermis in eyelid tissue. **E** Reactive lymphatic follicle with apoptotic cells in the surrounding lymph node. **F** Reactive lymphatic follicle with scattered apoptotic cells in the spleen. High-resolution images are provided in the supplementary materials (Supplementary Fig. 2A-F)

In the oral cavity, there was an ulceration in the epithelium, and in the adjacent epithelium, vacuolar degeneration of keratinocytes was observed. In the connective tissue below the ulceration area, there was moderate infiltration of inflammatory cells, with a dominance of lymphocytes and plasma cells (Fig. 2A). The epidermis of the muzzle was focally ulcerated. In the adjacent epidermis, keratinocytes were necrotic or swollen with signs of degeneration. In the area of ulceration, there was abundant infiltration of inflammatory cells, with neutrophils dominating the epidermis and lymphocytes, plasma cells, and macrophages in the underlying dermis (Fig. 2B-C). Examination of the eyelid revealed a hyperplastic and hyperkeratotic epidermis close to the conjunctiva, with moderate to occasionally abundant infiltration of inflammatory cells and a dominance of lymphocytes and plasma cells in the underlying dermis (Fig. 2D). Abundant reactive lymphatic follicles with germinal centers were observed in the lymph node. The lymph nodes contained numerous apoptotic cells and cavities that were believed to have contained apoptotic cells (Fig. 2E). The spleen contained many reactive lymphatic follicles with germinal centers and relatively extensive apoptosis (Fig. 2F).

### Real-time polymerase chain reaction of parapoxvirus and cervidpoxvirus

Samples from five reindeer calves were analysed for PPV and CvPV by qPCR (Table 1). Two of the calves were positive for PPV, with the lowest Ct values, indicating the highest viral load, in the samples from the eyelid and oral mucosa of Calf 5. Four calves were positive for CvPV, indicating that both viruses were present in the herd. Both viruses were detected in Calf 5.

### Whole-genome sequencing

A 143,682 bp sequence of the reindeer RDPV isolate 23-MIK191411 (hereafter referred to as reindeer RDPV; GenBank accession PV021565.1) was assembled directly from nucleic acid extracted from an oral mucosal swab of Calf 5 without virus isolation or cultivation. All, except for a 384 nt portion (see Methods), of the sequence was obtained by de novo assembly with read coverage above 500x. No sequences of CvPV were detected in the sample, indicating a lower viral load than that of PPV.

### Sequence comparison with RDPV HL953

**Fig. 3:**
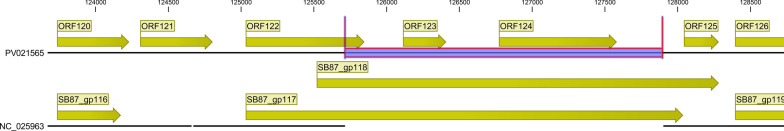
The region of the sequence alignment of parapoxvirus red deer (RDPV) HL953 (bottom) and reindeer RDPV isolate 23-MIK191411 (reindeer RDPV) (top), showing the insert region, with the insert marked in blue. Open reading frames (ORFs) are indicated with yellow bars with ORF numbers for the reindeer RDPV and with locus tags (SB87_gp###) for the RDPV HL953. The two sequences are marked by their GenBank accession numbers. At the left end, ORF120/SB87_gp116, where ORF120 is longer due to a mutation in the stop codon, is shown. The hypothetical protein ORF121 is not annotated in RDPV HL953. ORF122 and SB87_gp117 are putative GM-CSF/IL-2 inhibitory factors, where for ORF122, a large part is altered by the insert. ORF123 and ORF124 (the latter of which is a putative NF-κB inhibitor) are contained in the insert. For RDPV HL953 (without an insert), the corresponding putative GM-CSF/IL-2 inhibitory factor (SB87_gp117) has a large ORF overlap with SB87_gp118 — a feature uncharacteristic of poxviruses — which encodes a hypothetical protein. Finally, at the right end of the reindeer RDPV genome, ORF125 encodes a closely related but truncated version of SB87_gp118

The RDPV HL953, 139,981 bp in length, with the GenBank accession NC_025963.1, is the current reference sequence for the RDPV. The reindeer RDPV described in this study is closely related to RDPV HL953, and the following sections detail the genomic differences between the two isolates. The GC contents are 64.9% and 65.0% for reindeer RDPV and RDPV HL953, respectively, whereas the reindeer RDPV sequence is 3,701 bp longer than RDPV HL953. This difference is mainly due to a 2,187 bp insert in the right terminal third of the sequence (Fig. 3), and more of the right and left terminal regions are determined in reindeer RDPV, including parts of the inverted terminal repeats. All 130 ORFs predicted in RDPV HL953 are also present in the reindeer RDPV. In addition, using reindeer RDPV ORF numbering, two left terminal ORFs, ORF1 and ORF2, are predicted in reindeer RDPV, where ORF1 shares approximately 50% nucleotide sequence identity with ORF1s predicted in other PPVs. Three ORFs, ORF17, ORF30, and ORF121, carry the corresponding stop and start codons but were not predicted in RDPV HL953. A codon deletion in ORF17 relative to the sequence in RDPV HL953 provides some evidence that this ORF may be expressed. Similarly, ORF121 has a 4-codon insert relative to the unannotated sequence of RDPV HL953. The 2,187 bp insert introduces two ORFs, ORF123 and ORF124, with ORF124 tentatively encoding an NF-κB inhibitor. Thus, in total, 137 ORFs are predicted in reindeer RDPV. The amino acid sequences of the 130 commonly predicted ORFs are identical for 55 ORFs (42%) and have fewer than four amino acid differences for 115 ORFs (88%). Three ORFs, ORF25, ORF75, and ORF126, were identified as hypothetical proteins in RDPV HL953, but their sequence homology suggests that these ORFs are putative NF-κB inhibitors. Likewise, sequence homology suggests that ORF29, designated as a hypothetical protein in RDPV HL953, might be an ERK1/2 activator protein.

### Phylogenetic analysis

**Fig. 4:**
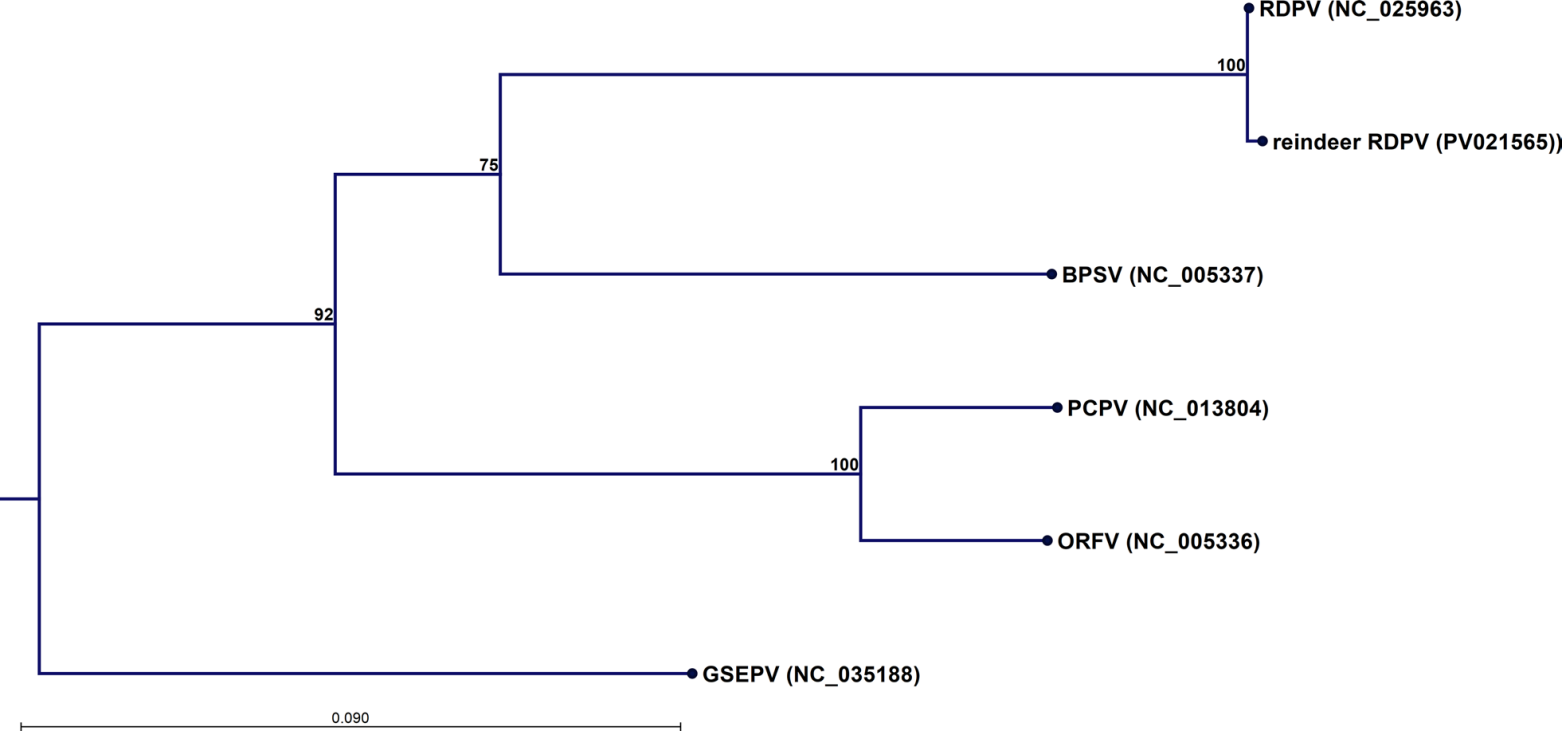
Maximum likelihood phylogenetic tree based on the amino acid sequence of the concatenated alignments of the ten parapoxvirus core proteins: poly-A polymerase catalytic subunit, DNA polymerase, RNA polymerase associated protein RP94, RNA polymerase subunit RPO132, IMV protein VP55, DNA topoisomerase type 1, EEV envelope phospholipase, Serine/Threonine kinase, DNA helicase, and RNA helicase for isolate 23-MIK191411 (reindeer RDPV) and various other parapoxviruses (BPSV = bovine papular stomatitis virus, ORFV = orf virus, PCPV = pseudocowpox virus, GSEPV = seal parapoxvirus, and RDPV = parapoxvirus red deer)

Figure 4 presents the maximum likelihood phylogeny based on the concatenation of ten proteins from the conserved PPV core genome and shows that the reindeer RDPV is highly similar to RDPV HL953. The RDPVs form a clade together with their closest relative BPSV, separate from the clade formed by PCPV and ORFV. The most genetically divergent PPV is the GSEPV.

**Fig. 5:**
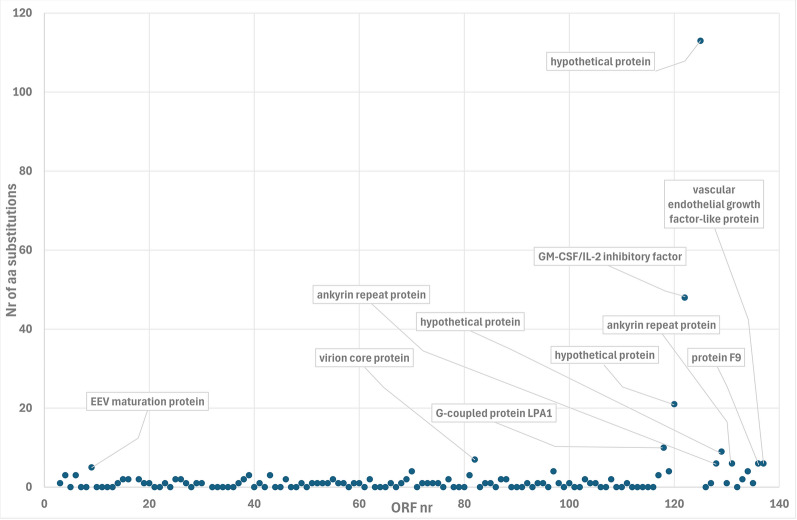
The number of amino acid substitutions in parapoxvirus red deer from the reindeer isolate 23-MIK191411 (reindeer RDPV) relative to RDPV HL953 for each common open reading frame (ORF) along the sequence. Open reading frames with five or more amino acid alterations are indicated. Differences in length are considered alterations except when they are caused by alternative start codons available in both sequences

In Fig. 5, the number of amino acid substitutions per ORF in reindeer RDPV relative to RDPV HL953 is shown along the sequence for each of the 130 common ORFs. The number of substitutions is highest at the termini of the sequence, especially at the right terminal. ORFs with five or more substitutions are indicated. The only ORFs reaching this level at the left terminal and central regions are putatively encoding an extracellular enveloped virus maturation protein and a virion core protein homologous to the vaccinia virus protein A4, respectively. At the right terminal region, the following ORFs with five or more amino acid substitutions putatively encode two of three ankyrin repeat proteins in this region: a vascular endothelial growth factor-like protein, a protein homologous to the vaccinia virus protein F9, a GM-CSF/IL-2 inhibitory factor, and a G-coupled protein LPA1, in addition to three hypothetical proteins. The largest change is caused by the insert, which cleaves the ORF of a 191 amino acid hypothetical protein present in RDPV HL953, and at the right terminus of this protein, an ORF encoding a 79 amino acid hypothetical protein (ORF125) is left. At the left end of the insert, the GM-CSF/IL-2 inhibitory factor is strongly affected, leading to the substitution of 48 amino acids relative to the RDPV HL953 counterpart. Moreover, the hypothetical protein encoded by ORF120 undergoes substantial alteration due to a mutation of a stop codon, which leads to the addition of 19 amino acids, partly constituted by a duplication of an SSSSSSSL-motif and the addition of a positively charged RRRK tail.

### Open reading frames introduced by the insert

The two ORFs on the insert, ORF123 and ORF124, as well as the truncated ORF125, have homologous counterparts in other PPVs. For example, for BPSV, PCPV, and ORFV with accessions PP565898, JF792399, and NC_005336, ORF118-120, with accessions WZD65462-64, AEL20653-55, and NP_957895-7, respectively, have BLASTp (Basic Local Alignment Search Tool for proteins) amino acid sequence identities in the range of 35.7–57.14% with a query (other PPVs) coverage in the range of 67–100%, except for the truncated ORF125, which naturally has a lower query coverage in the range of 22–36%. The GSEPV, with accession NC_035188, also has counterparts to ORF123 and ORF124, namely, the hypothetical proteins with locus tags CGV_gp196-7, which have sequence identities of 43.6 and 50.4%, respectively, with corresponding query coverages of 73 and 77%. However, GSEPV appears to lack an ORF producing a protein homologous to ORF125 (ORF120 in the other PPVs). Since there were simultaneous cases of CvPV infections in the herd and weakly positive PCR results for CvPV in Calf 5 (Table 1), the insert was initially hypothesized to have originated from this CvPV. Recently, a partial genome of CvPV was reported by Nymo et al. 2025 [21]. Sequence comparison between the insert and the CvPV genome revealed that the insert did not originate from this CvPV genome (unpublished data). This finding is also consistent with the fact that the ORFs of the insert only have sequence homology with other available PPVs. Attempts to detect recombination using RDP5 [33] with representative donor PPV genomes from RDPV, BPSV, ORFV, PCPV, and GSEPV were unsuccessful.

## Discussion

This study reports the first detection of RDPV in reindeer, suggesting a host shift from red deer.

Clinical examinations were performed on five out of ten affected reindeer calves. Real-time PCR analysis detected PPV in two of the calves and CvPV in four calves. Both viruses were identified in calf 5, where no contigs were classified as CvPV, indicating that this virus was present below the detection limit of the NGS run, consistent with the high Ct-value of 34.9. The affected calves in the herd displayed clinical signs similar to those of CE [7, 16, 19], characterized by crusts and lesions on the muzzle, lips, and oral mucosa (Fig. 1 and Supplementary Fig. 1). Histopathology confirmed nonspecific hyperkeratosis with infiltration of inflammatory cells in the cutis and subcutis, which is not indicative of a specific pathogen (Fig. 2). Similar histopathological findings were reported in an experimental infection study of ORFV in reindeer [34]. In addition, most PPV lesions in reindeer are mainly observed in the oral mucosa and not in the skin around the mouth, including secondary bacterial infections [7, 19, 34].

Therefore, the exact cause of the lesions remains unclear, as several factors could have contributed. Herders suspected insect harassment, especially around the eyelids, as a possible trigger. Additionally, the recently described CvPV, detected in four calves with clinical signs, causes lesions in the skin of the eyelids and external genitalia [21]. It is therefore plausible that both the reindeer RDPV and CvPV contributed to the observed clinical signs. Insects may contribute to disease transmission, as demonstrated in lumpy skin disease, a capripoxvirus infection of cattle, where flies, mosquitoes, midges, and ticks act as vectors [35]. Additionally, rodents are reservoirs for other poxviruses [36], and their role in reindeer poxvirus ecology cannot be excluded. Thus, the potential involvement of insects and rodents in the transmission and spread of reindeer RDPV and CvPV warrants further investigation. In addition, the intensification of reindeer husbandry due to pastureland loss, leading to supplementary feeding, increased animal density, and stress levels, might facilitate the spread of infectious diseases [24]. Regular and sporadic disease outbreaks of CE have been reported in Finland since the early 1990s, including several severe epidemics with high mortality, thought to be associated with supplementary feeding, severe winter weather conditions, introduction of a new virus, and secondary bacterial infections linked to fatal cases [7, 14]. Routes of transmission of PPVs to reindeer are thought to be either via direct contact with sheep, goats, and cattle, or indirectly through sharing pastures, corrals, transport vehicles, and roughage, as PPVs are highly stable in the environment [7, 37]. Remnants of fencing from former red deer farms persist within reindeer pastures used by the reindeer in this study and may have served as an indirect route of transmission, in addition to potentially shared pastures and direct contact when the farms were still operational. Moreover, climate change may influence the ecology of potential vectors and reservoirs, geographic distribution, and temporal persistence of various pathogens [38]. Together, these factors may increase reindeer susceptibility to infectious pathogens, such as reindeer RDPV and CvPV, resulting in enhanced pathogen shedding and more severe clinical manifestations. Transmission of parapoxvirus from cervids to humans during slaughter has been reported [39, 40]. Even though RDPV was not the cause in these cases, the zoonotic potential of PPVs poses a potential occupational health risk for humans in contact with reindeer.

The phylogenetic comparison of ten concatenated conserved genome core proteins (Fig. 4) shows that reindeer RDPV is highly similar to the RDPV HL953 [8]. The resulting overall tree topology is concordant with the one previously obtained using 47 conserved proteins [41]. Only eleven out of the 130 common ORFs presented more than 5 amino acid alterations, including changes in length (Fig. 5). The majority of these eleven ORFs are located at the right terminal region of the genome. The terminal regions of poxviruses contain genes that affect host range and pathogenesis [42, 43]. In a genomic comparison of ORFV and BPSV [42], ORF80 (ORF82 in the present reindeer RDPV numbering, c.f. Figure 5), which is a homologue of Vaccinia virus A4L and encodes a virion core protein, was pinpointed as a gene of significant variability. The corresponding gene in the reindeer RDPV is indeed one of the ORFs most different from RDPV HL953 (Figs. 4 and 5). This gene was also shown to be particularly variable in orthopoxvirus genomes [43]. Similarly, right terminal ankyrin repeat proteins have also been shown to be particularly variable [42, 43], a pattern that was likewise observed in the present study (Fig. 5). Ankyrin repeat proteins are typical of poxviruses and are implicated in host interactions [44]. ORF118 is also markedly different from its counterpart in RDPV HL953, with ten amino acid substitutions (Fig. 5). The homologue of ORF118 in ORFV, ORF113, has been shown to interact with the G protein-coupled receptor lysophosphatidic acid receptor 1 (LPA1) to increase p38 phosphorylation, which strongly promotes virus replication in infected cells [45]. The putative GM-CSF/IL-2 inhibitory factor (ORF122) combats the host immune response, and ORF136 is a homologue of Vaccinia virus protein F9, which is known to be required for cell entry [46]. The final ORF at the right terminal (ORF137), with six amino acid substitutions relative to RDPV HL953 (Fig. 5), encodes a viral analogue to vascular endothelial growth factor (VEGF), which also varies greatly between ORFV and BPSV [42]. Parapoxviral VEGF has been shown to be a pathogenicity factor associated with vascularization and lesion proliferation [47], which are likely to be host dependent as well. Thus, the vast majority of the ORFs with a comparatively large number of substitutions relative to RDPV HL953 encode proteins involved in host interactions and pathogenesis.

The insert contains two ORFs (ORF123 and ORF124). Both of these ORFs have homologues located in approximately the same genomic region in four other PPV species (ORF118–19 in ORFV, PCPV, and BPSV, locus tags CGV03_gp106–7 in GSEPV) but are absent from RDPV HL953. Interestingly, in a PCPV infecting reindeer, the loss of a 5,431 bp sequence encompassing ORF116–121 was observed after serial passage in cell culture [48]. Notably, this region includes the corresponding region of the whole insert in the present study. This observation suggests that, rather than an insert in the sequence obtained in the present study, there is a deletion in RDPV HL953, since the RDPV HL953 genome sequence was obtained from a virus collected after five passages in cell culture. However, the large alteration of the GM-CSF/IL-2 inhibitory factor (ORF122) at the left terminal of the purported insert and the truncation of ORF125 at the right terminal, which is only 79 amino acids in reindeer RDPV but 139 amino acids in BPSV (PP565898), 194 amino acids in ORFV (NC_005336), and 204 amino acids in PCPV (JF792399), strongly indicate that there has been an insertion in the reindeer RDPV. Notably, there may still be a deletion in RDPV HL953, which led to the loss of the ORFs corresponding to ORFs 123–24. The sequence identity of ORF123 to the other PPVs ranges from 43.6–46.5% over approximately 70% of the target, and that of the putative NF-κB inhibitor (ORF124) is 50–57.1% over 70–97% of the target. This low sequence identity suggests that the insert may derive from a previously unknown PPV species native to reindeer, rather than from any currently described PPVs. This is consistent with Tryland et al. (2019) [18], who found no evidence of a specific reindeer ORFV circulating in Fennoscandia based on GIF gene phylogeny. Attempts to detect a recombination event with currently characterized PPVs (RDPV, BPSV, PCPV, ORFV, and GSEPV) as potential donors using RDP5 were unsuccessful (data not shown). This is not unexpected since the sequence homology between the insert and any of these PPVs is very low.

The novel reindeer RDPV thus systematically displays a comparatively large number of amino acid alterations relative to the RDPV HL953 isolate in proteins involved in host interactions and pathogenesis. In addition, a unique insert was found that could play a key role in regulating the immune response. These observations suggest that the virus has adapted to the reindeer host for some time. The insert carries two ORFs, both with weak sequence homology to putative ORFs in all other currently known PPVs, except RDPV, at roughly the same genomic location. A currently unknown PPV likely recombined with RDPV, adapted, and established itself as a novel PPV in reindeer. This could indicate that previous red deer populations in the herding district introduced the RDPV to the reindeer herd in the same area, enabling the RDPV to recombine with an unknown PPV already circulating in the reindeer herd. These events may significantly affect reindeer health, particularly in conjunction with climate change and the intensification of reindeer husbandry, which may facilitate the emergence and spread of disease. The potential zoonotic risk underscores the importance of continuous health surveillance to monitor emerging diseases and guide management strategies.

## Conclusions

This is the first report of a full-genome-sequenced RDPV in reindeer associated with clinical lesions, and the first detection of RDPV in a species other than red deer. The clinical signs were indistinguishable from previously reported lesions caused by other PPVs in reindeer as well as CvPV, which highlights the challenges in determining the causative agent of an outbreak based solely on clinical observations, and emphasizes the need for molecular methods such as sequencing and qPCR. The novel reindeer RDPV shows evidence of long-term adaptation to reindeer, including unique genetic changes likely resulting from recombination with an unknown PPV. This finding suggests cross-species transmission, possibly involving red deer, and highlights the risk of emerging diseases in reindeer, underscoring the importance of continued health surveillance.

## Supplementary Information

Supplementary Figure

Supplementary Figure 2A

Supplementary Figure 2B

Supplementary Figure 2C

Supplementary Figure 2D

Supplementary Figure 2E

Supplementary Figure 2F

## Data Availability

The complete genome of the parapoxvirus red deer presented in this paper has been deposited in GenBank with the accession number PV021565.1. The authors confirm that all supporting data, code, and protocols have been provided within the article or through supplementary data files.

## Abbreviations

**PPV**: Parapoxvirus
**ORFV**: Orf virus
**BPSV**: Bovine papular stomatitis virus
**PCPV**: Pseudocowpox virus
**GSEPV**: Seal parapoxvirus
**RDPV**: Parapoxvirus red deer
**CE**: Contagious ecthyma
**CvPV**: Cervidpoxvirus
**ORF**: Open reading frame
