# Supplementary material for: First report of a parapoxvirus red deer infection in reindeer (Rangifer tarandus tarandus): clinical presentation and full-genome characterization
Source: Virol J. 2025 Dec 31;23:87. doi: 10.1186/s12985-025-03046-5 (PMC13040740; doi:10.1186/s12985-025-03046-5)
Supplement: Supplementary file 1 — Supplementary Figure [file 12985_2025_3046_MOESM1_ESM.pdf]

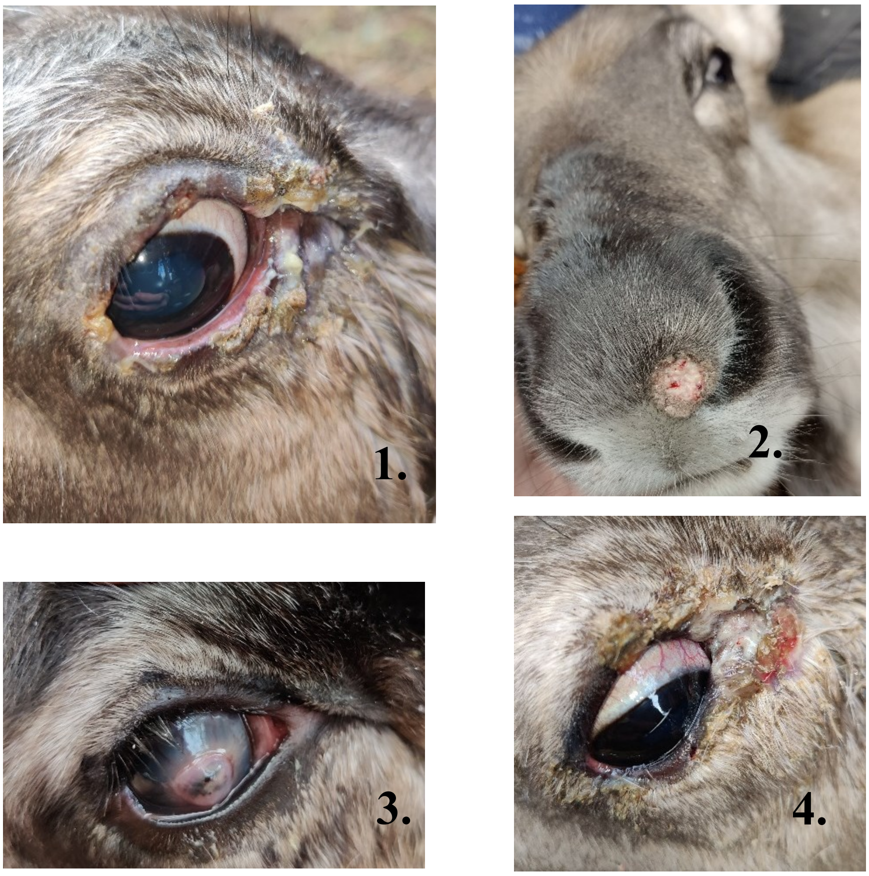

Supplementary Fig. 1. Crusty lesions on the eyelids, with purulent discharge and epiphora, in Calves 1 and 4; focal lesions on the muzzle and dorsolateral on the nasal bridge of Calf 2, without eyelid involvement; and a corneal ulcer with periocular alopecia in Calf 3, presumably following healed eyelid lesions. The calves were among five isolated semi-domesticated reindeer (*Rangifer tarandus tarandus*) originating from a gathering of approximately 150 reindeer in September 2023, in Norrbotten County, Sweden. Photo: Veronica Lengquist, SLU.
