# Supplementary figures and images for: First report of a parapoxvirus red deer infection in reindeer (Rangifer tarandus tarandus): clinical presentation and full-genome characterization
Source: Virol J. 2025 Dec 31;23:87. doi: 10.1186/s12985-025-03046-5 (PMC13040740; doi:10.1186/s12985-025-03046-5)

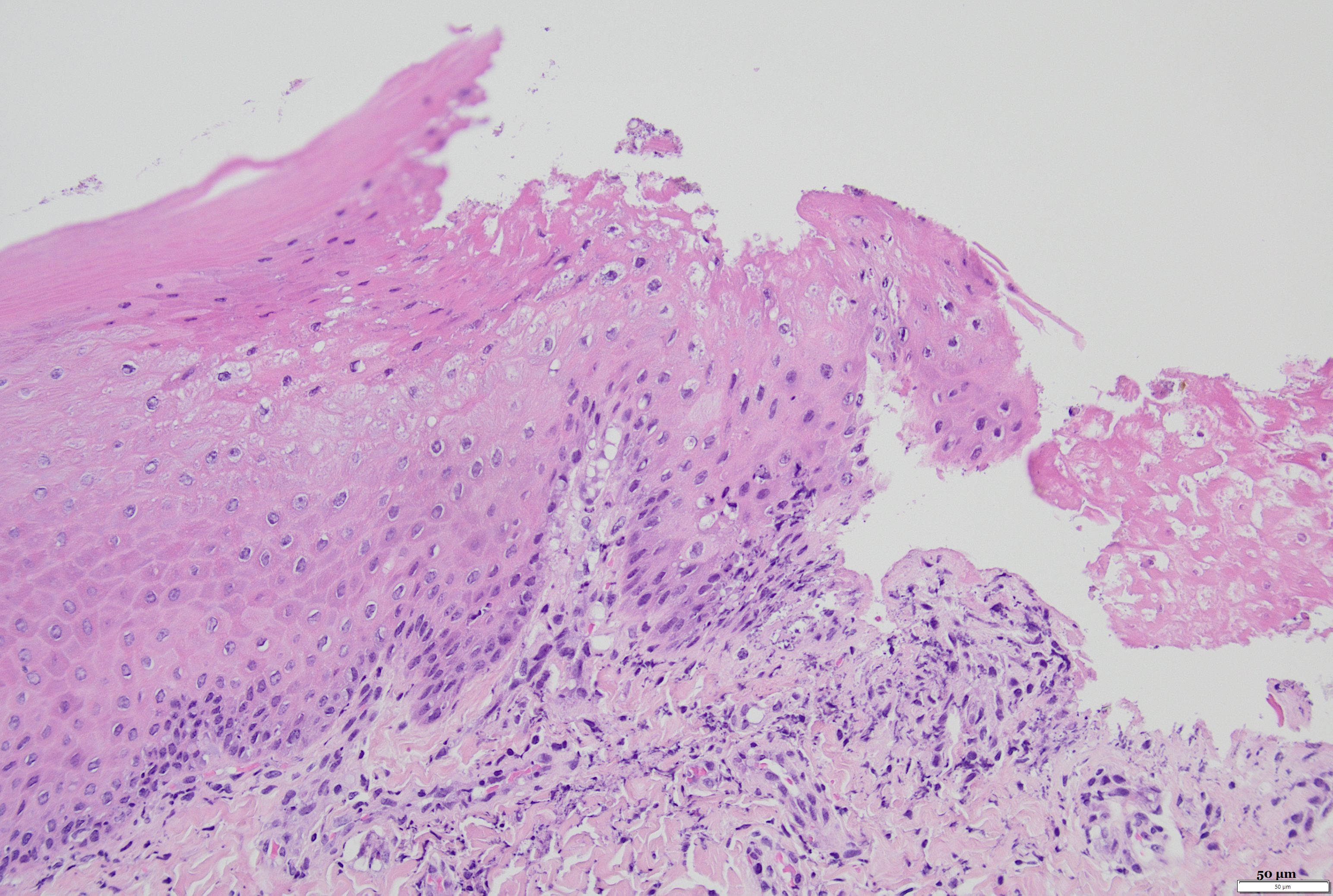

Supplement: Supplementary file 2 — Supplementary Figure 2A [file 12985_2025_3046_MOESM2_ESM.tif]

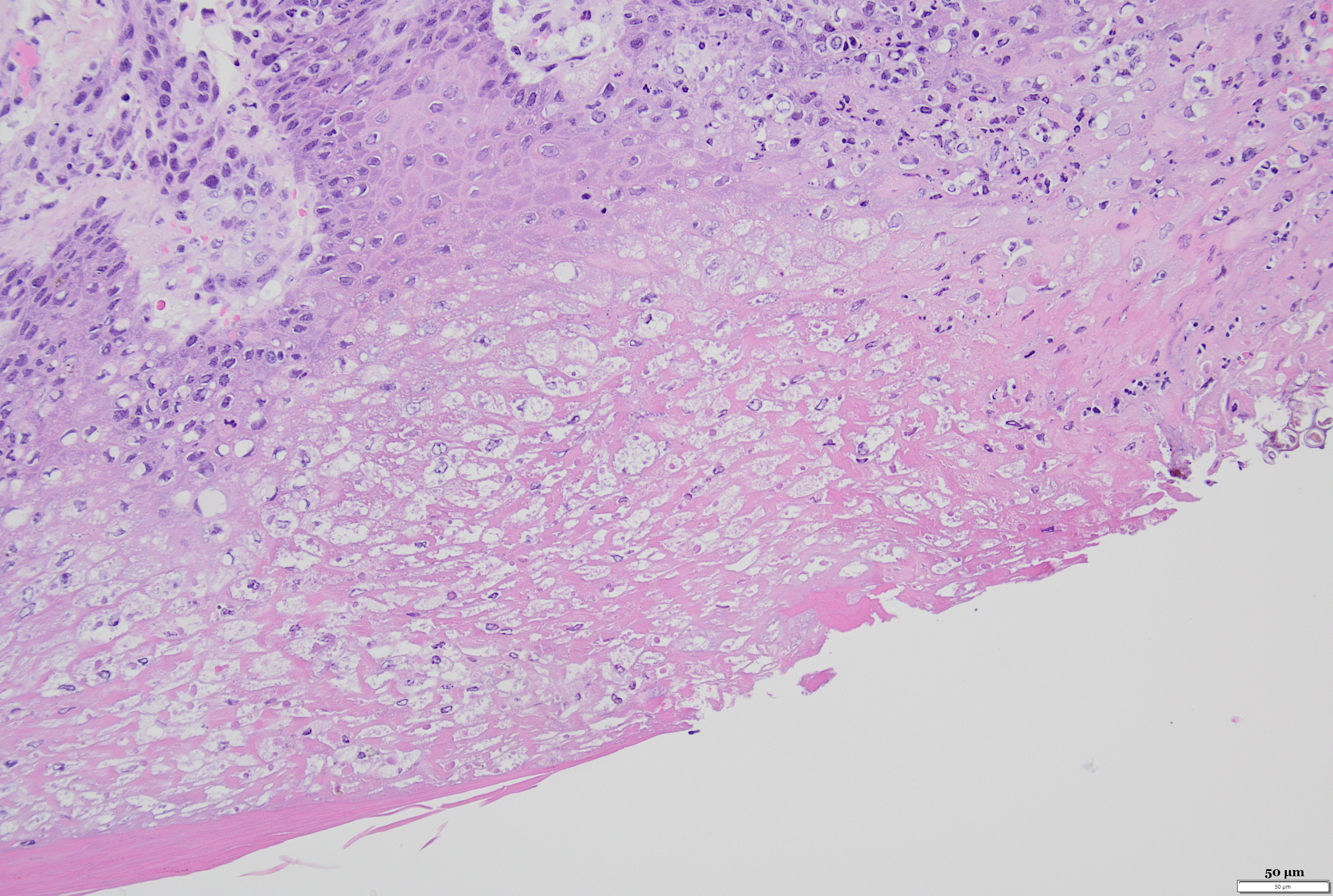

Supplement: Supplementary file 3 — Supplementary Figure 2B [file 12985_2025_3046_MOESM3_ESM.tif]

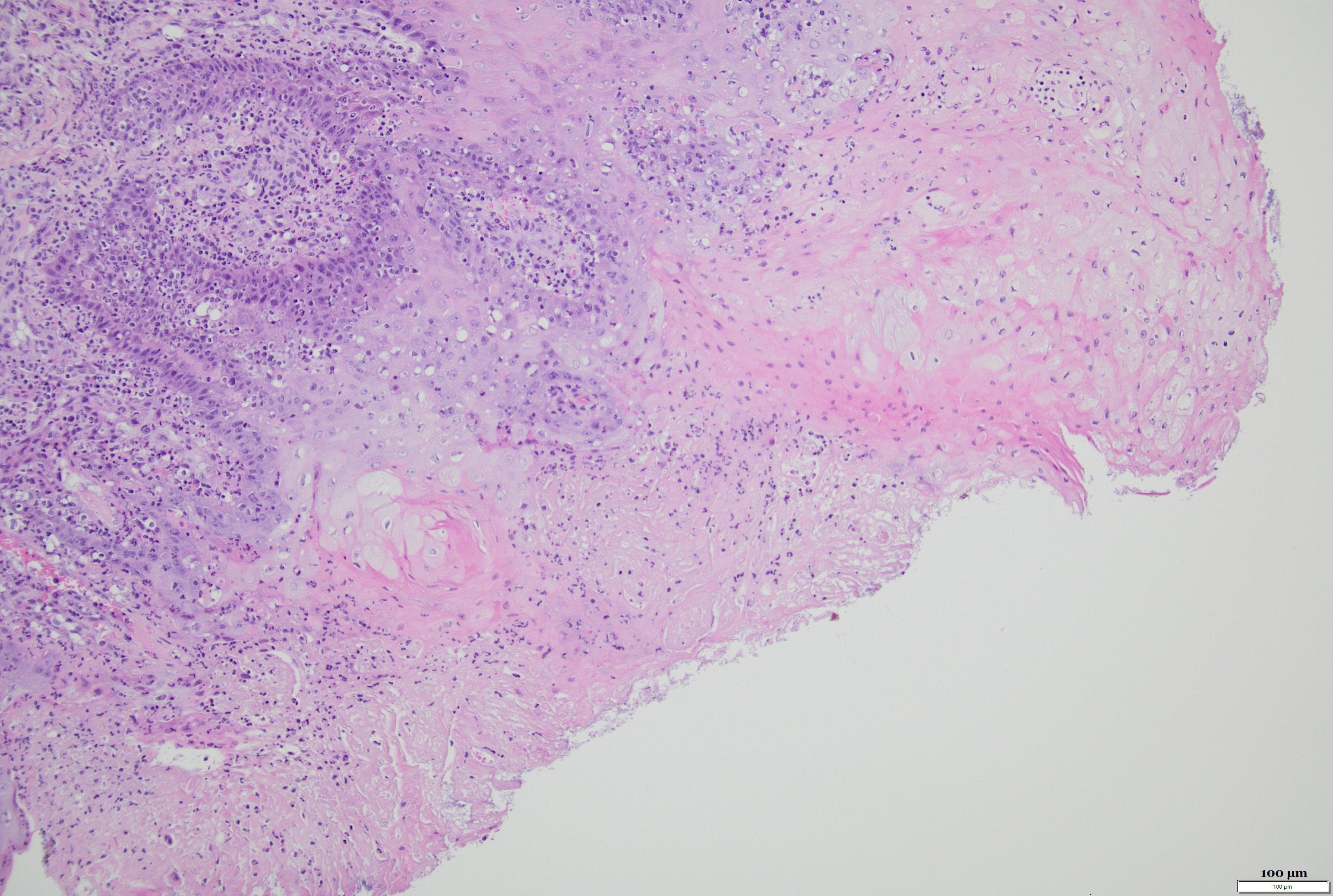

Supplement: Supplementary file 4 — Supplementary Figure 2C [file 12985_2025_3046_MOESM4_ESM.tif]

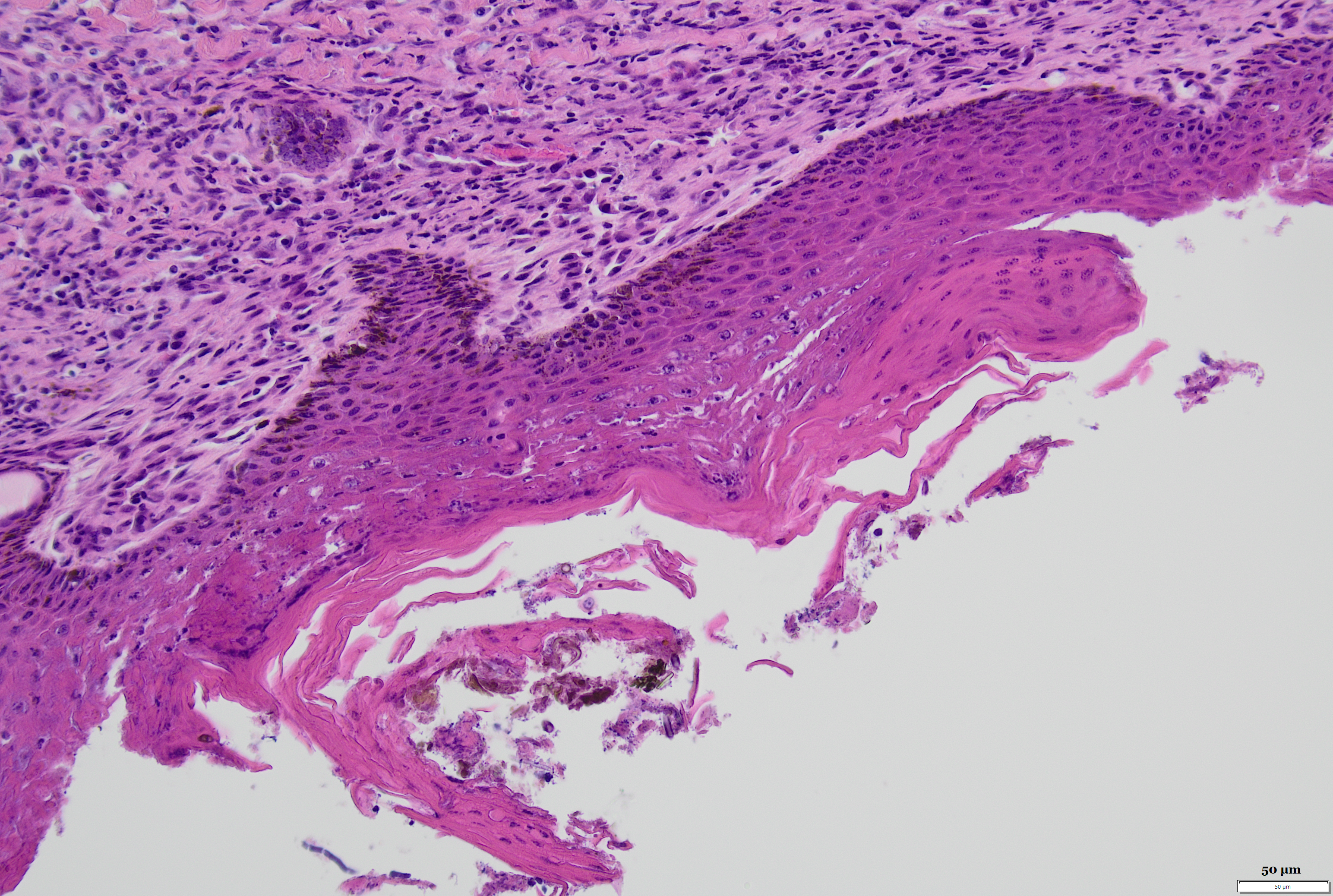

Supplement: Supplementary file 5 — Supplementary Figure 2D [file 12985_2025_3046_MOESM5_ESM.tif]

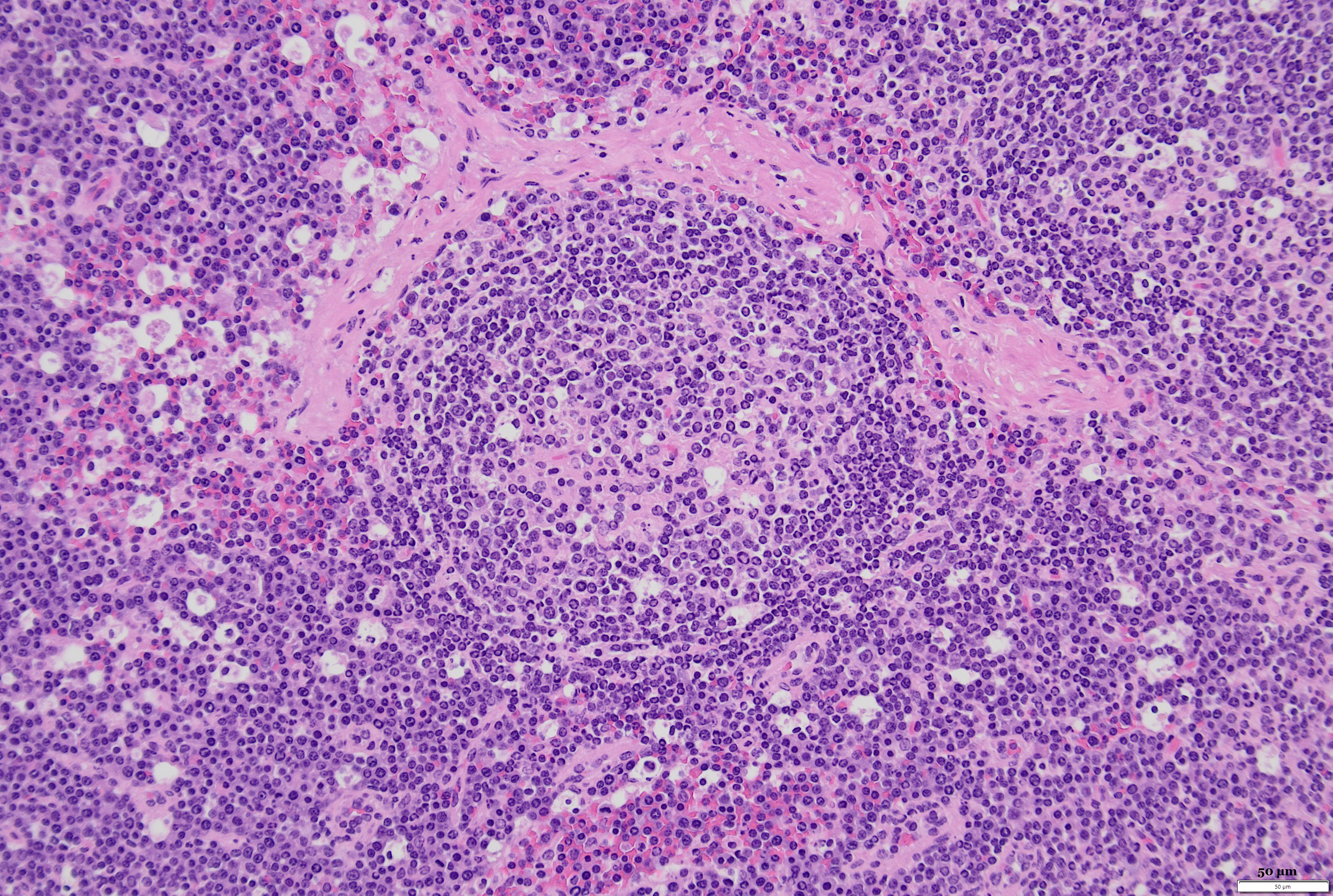

Supplement: Supplementary file 6 — Supplementary Figure 2E [file 12985_2025_3046_MOESM6_ESM.tif]

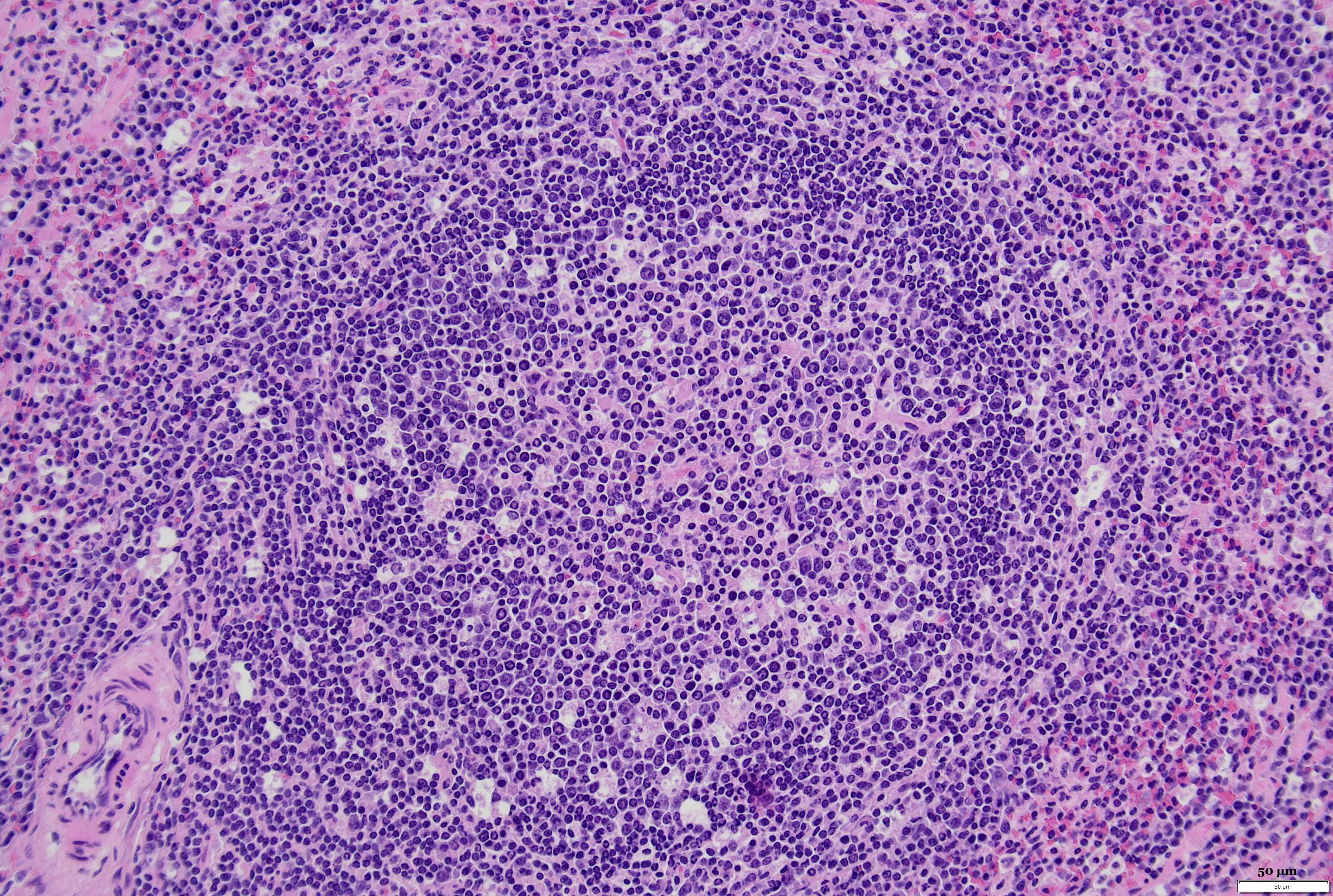

Supplement: Supplementary file 7 — Supplementary Figure 2F [file 12985_2025_3046_MOESM7_ESM.tif]
